# Cu-Contamination-Free Hybrid Bonding via MoS_2_ Passivation Layer

**DOI:** 10.3390/nano15201600

**Published:** 2025-10-21

**Authors:** Hyunbin Choi, Kyungman Kim, Sihoon Son, Dongho Lee, Seongyun Je, Jieun Kang, Sunjae Jeong, Doo San Kim, Minjong Lee, Jiyoung Kim, Taesung Kim

**Affiliations:** 1 Department of Semiconductor Convergence Engineering, Sungkyunkwan University, Suwon 16419, Gyeonggi-do, Republic of Korea; hyunvin99@skku.edu; 2 SKKU Advanced Institute of Nanotechnology (SAINT), Sungkyunkwan University, Suwon 16419, Gyeonggi-do, Republic of Korea; kkm8548@naver.com (K.K.); jemina01@skku.edu (S.S.); mentos2ro@gmail.com (S.J.); 3 Department of Nano Science and Technology, Sungkyunkwan University, Suwon 16419, Gyeonggi-do, Republic of Korea; 4 School of Mechanical Engineering, Sungkyunkwan University (SKKU), Suwon 16419, Gyeonggi-do, Republic of Korea; dongholee@skku.edu; 5 School of Advanced Materials Science and Engineering, Sungkyunkwan University (SKKU), Suwon 16419, Gyeonggi-do, Republic of Korea; wldms8999@naver.com; 6 Department of Display Engineering, Sungkyunkwan University (SKKU), Suwon 16419, Gyeonggi-do, Republic of Korea; sunash@skku.edu; 7 Department of Materials Science and Engineering, The University of Texas at Dallas, Richardson, TX 75080, USA; doosan.kim@utdallas.edu (D.S.K.);; 8 Department of Electrical and Computer Engineering, The University of Texas at Dallas, Richardson, TX 75080, USA; minjong.lee@utdallas.edu

**Keywords:** hybrid bonding, passivation layer, TMD, PECVD

## Abstract

Hybrid bonding technology has emerged as a critical 3D integration solution for advanced semiconductor packaging, enabling simultaneous bonding of metal interconnects and dielectric materials. However, conventional hybrid bonding processes face significant contamination challenges during O_2_ plasma treatment required for OH group formation on SiCN or the other dielectric material surfaces. The aggressive plasma conditions cause Cu sputtering and metal migration, leading to chamber and substrate contamination that accumulates over time and degrades process reliability. In this work, we present a novel approach to address these contamination issues by implementing a molybdenum disulfide (MoS_2_) barrier layer formed through plasma-enhanced chemical vapor deposition (PECVD) sulfurization of Mo films. The ultrathin MoS_2_ layer acts as an effective barrier preventing Cu sputtering during O_2_ plasma processing, thereby eliminating chamber contamination, and it also enables post-bonding electrical connectivity through controlled Cu filament formation via memristive switching mechanisms. When voltage is applied to the Cu-MoS_2_-Cu structure after hybrid bonding, Cu ions migrate through the MoS_2_ layer to form conductive filaments, establishing reliable electrical connections without compromising the bonding interface integrity. This innovative approach successfully resolves the fundamental contamination problem in hybrid bonding while maintaining excellent electrical performance, offering a pathway toward contamination-free and high-yield hybrid bonding processes for next-generation 3D-integrated devices.

## 1. Introduction

The semiconductor industry continues to face increasing demands for higher performance and greater integration density, driving the development of advanced three-dimensional packaging technologies [1]. Among these technologies, hybrid bonding has emerged as a key enabler for fine pitch interconnections, offering simultaneous metal-to-metal and dielectric-to-dielectric bonding at the wafer level [2,3]. Unlike conventional solder-based interconnects, hybrid bonding provides superior electrical performance and enables sub-micrometer pitch scaling essential for next-generation heterogeneous integration applications [2,4]. The hybrid bonding process relies on plasma surface activation to create hydroxyl groups on dielectric surfaces, particularly for silicon carbonitride (SiCN) systems that have gained popularity due to their excellent Cu diffusion barrier properties and mechanical strength [5,6]. The oxygen plasma treatment is crucial for achieving strong dielectric bonds through hydroxyl group formation and subsequent covalent bond formation during annealing [7,8]. However, this process introduces significant contamination challenges that have become increasingly problematic as device dimensions continue to scale down and due to process reliability.

**Figure 1:**
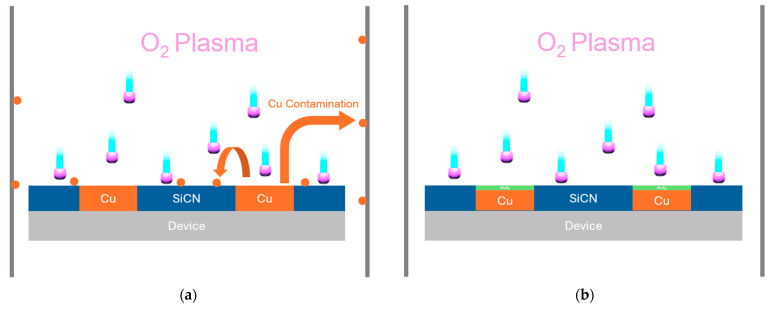
(**a**) A scheme in which the absence of a passivation layer leads to contamination of both the chamber and the wafer; (**b**) MoS_2_ blocks Cu sputtering, and the contamination is reduced.

During O_2_ plasma treatment, the high-energy plasma environment causes substantial sputtering of Cu and other metallic materials from interconnect structures [9,10]. This sputtered material accumulates on chamber walls and contaminates subsequent wafers, leading to several critical issues, including non-uniform plasma conditions due to changing chamber wall properties, cross-wafer contamination, particle generation that can cause device failures, and reduced process reproducibility over time (Figure 1a) [11,12]. For fine-pitch devices, even small amounts of Cu contamination can result in electrical shorts or reliability failures [13]. Traditional approaches to mitigate contamination have focused on chamber cleaning protocols, alternative plasma chemistries, or modified process conditions [14,15]. However, these solutions often involve trade-offs in bonding quality, increased process complexity, or higher thermal budgets that may not be compatible with temperature-sensitive devices [16]. The need for a fundamental solution that addresses contamination at its source while maintaining bonding performance has become increasingly urgent.

Transition metal dichalcogenides (TMDs), particularly transition metal dichalcogenides like MoS_2_, have demonstrated exceptional properties such as diffusion barriers and functional layers in semiconductor applications [17,18]. MoS_2_ exhibits superior barrier performance against Cu diffusion compared to conventional Ta/TaN systems, with effectiveness maintained even at thicknesses below 2 nm (Figure 1b) [19,20]. Additionally, TMDs’ layered structure enables controllable ion migration and memristive behavior, opening new possibilities for functional integration [21,22]. Recent advances in plasma-enhanced chemical vapor deposition (PECVD) have enabled precise control of MoS_2_ film properties while maintaining compatibility with back-end-of-line processing [23,24]. This study identified the process conditions for sulfurization PECVD that avoid adverse effects on the underlying Cu metal during MoS_2_ formation, thereby maintaining the electrical conductivity and structural integrity of the metal interconnects. Furthermore, the memristive properties of Cu-MoS_2_-Cu systems have been extensively demonstrated, showing reliable switching through Cu ion migration and filament formation [25,26].

This work introduces a novel approach that leverages MoS_2_’s dual functionality as both a contamination barrier during plasma processing and an active switching medium for post-bonding electrical connectivity. By sulfurizing ultrathin MoS_2_ layers on Cu trenches prior to hybrid bonding, we create a protective barrier that prevents Cu sputtering during O_2_ plasma treatment while enabling subsequent electrical connection through controlled Cu filament formation. When voltage is applied across the Cu-MoS_2_-Cu structure after bonding, Cu ions migrate through the MoS_2_ layer along defect sites, forming conductive filaments that establish electrical connectivity [27,28,29]. This approach offers complete elimination of Cu contamination during plasma processing, maintenance of clean chamber conditions for consistent process control, reliable electrical connectivity through controlled switching mechanisms, and potential for integration of memory and logic functions within the interconnect structure. This work establishes a foundation for contamination-free hybrid bonding while opening new avenues for functional integration in advanced semiconductor packaging applications and Monolithic 3D advanced technology [30].

## 2. Results and Discussion

**Figure 2:**
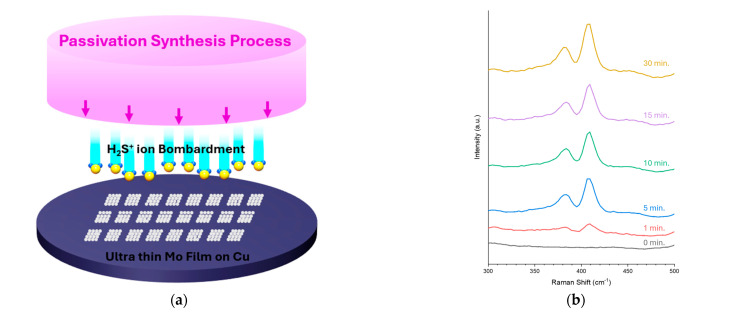

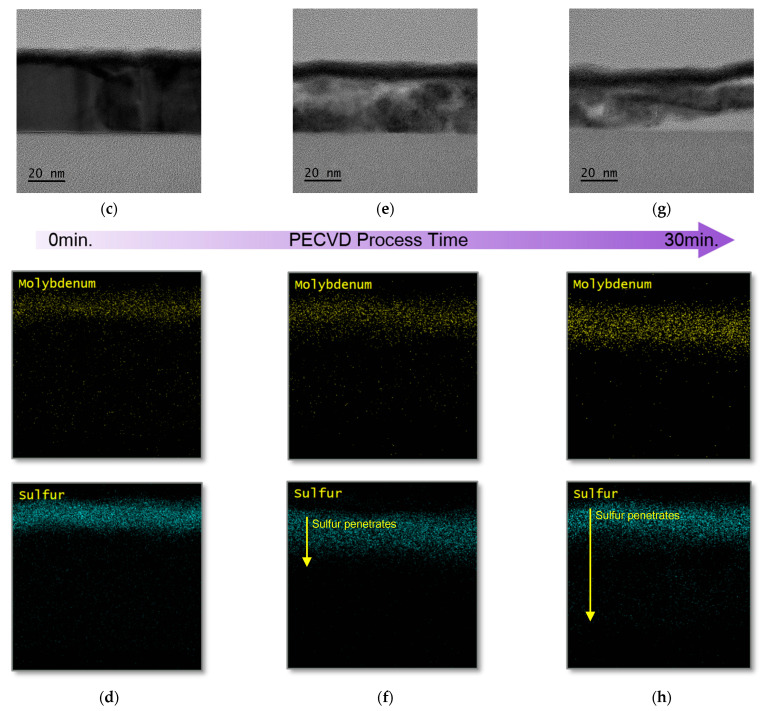
Plasma-assisted synthesis and time-dependent characterization of MoS2 thin films. (**a**) Schematic of MoS2 thin-film synthesis via a PECVD process; (**b**) Raman single spectrum data follow as an increase in PECVD process time; (**c**,**e**,**g**) cross-sectional TEM images acquired at increasing process times; (**d**,**f**,**h**) corresponding EDS elemental maps from the cross-sectional regions, revealing progressive sulfur penetration with longer process durations.

The passivation layer of Cu contamination during O_2_ plasma treatment for dielectric OH group formation using MoS_2_ was systematically implemented as illustrated in Figure 2a and the process flow can be found in Figure S1 of the Supplementary Materials. The fabrication process commenced with the formation of trenches in SiCN/Si substrates through Ar/CF_4_ capacitively coupled plasma (CCP) dry etching, providing precise dimensional control and vertical sidewall profiles essential for subsequent metallization steps. Following trench formation, electron beam evaporation was employed to sequentially deposit Cu for gap-fill and an ultra-thin Mo layer with precisely controlled thickness of 1.2 nm. The choice of electron beam deposition ensured conformal coverage and superior step coverage characteristics compared to conventional sputtering techniques, critical for maintaining uniform barrier layer thickness across complex three-dimensional topographies. The following sulfurization process was conducted using plasma-enhanced chemical vapor deposition (PECVD) with Ar + H_2_S plasma treatment at 150 °C, representing a significant advantage for back-end-of-line (BEOL) compatibility. This low thermal budget approach ensures compatibility with temperature sensitive interlayer dielectrics and organic substrates commonly employed in advanced packaging applications, while maintaining the integrity of underlying device structures [31,32]. The controlled sulfurization process specifically targets the surface Mo layer through H_2_S^+^ ion bombardment, facilitating the conversion of metallic molybdenum to molybdenum disulfide through the reaction: Mo + H_2_S → MoS_2_ + H_2_ [23,24]. To establish optimal process parameters and understand the kinetics of MoS_2_ formation, comprehensive Raman spectroscopic analysis was performed as a function of sulfurization time. The characteristic vibrational modes of MoS_2_, specifically the E^1^_2g_ peak at 383 cm^−1^ (corresponding to in-plane Mo-S stretching) and A_1g_ peak at 408 cm^−1^ (associated with out-of-plane S-Mo-S bending), serve as definitive fingerprints for crystalline MoS_2_ formation [33]. At the initial stage (0 min), the absence of these characteristic peaks confirmed that no MoS_2_ formation had occurred in the as-deposited Mo film. Progressive increase in sulfurization time revealed systematic enhancement of both peak intensities, indicating gradual conversion of the Mo precursor to crystalline MoS_2_. Notably, significant MoS_2_ formation was observed after 5 min of treatment, as evidenced by the emergence of well-defined E^1^_2g_ and A_1g_ vibrational modes with peak intensity ratios consistent with high-quality polycrystalline MoS_2_ (Figure 2b). Extended processing beyond 5 min showed negligible returns in terms of crystalline quality improvement. However, MoS_2_ formation was achieved at approximately 30 min, as indicated by peak sharpening characteristic of improved crystalline order. Despite this, extended processing times present significant risks to the underlying metallization integrity due to the penetration of high-energetic H_2_S^+^ ions through the thin Mo barrier layer. Cross-sectional transmission electron microscopy (TEM) analysis coupled with energy-dispersive X-ray spectroscopy (EDS) mapping provided critical insights into the depth dependent sulfurization behavior and potential Cu layer degradation (Figure 2c–h). At the optimized 5 min processing time, TEM imaging revealed successful conversion of only the uppermost Mo layer to MoS_2_, with preservation of the underlying Cu metallization integrity. In this sulfurization remained confined to the intended Mo barrier region, as confirmed by sulfur distribution mapping showing minimal penetration into the Cu layer. In contrast, extended processing times of 15 and 30 min demonstrated progressive Cu layer sulfurization and structural degradation. At 15 min, EDS analysis revealed significant sulfur penetration into the Cu layer, which compromises electrical conductivity and mechanical integrity [34]. The 30 min treatment resulted in severe Cu layer damage, including delamination, void formation, and complete loss of structural integrity in localized regions. This degradation occurs through the formation of volatile copper sulfide compounds and differential thermal expansion coefficients between the sulfurized and metallic regions [20]. Based on these comprehensive characterization results, the optimal sulfurization time of 5 min was established as the ideal compromise between achieving complete 5 nm MoS_2_ barrier formation and preserving underlying Cu metallization integrity. This processing window ensures that energetic H_2_S^+^ ions successfully convert the surface Mo layer to crystalline MoS_2_ while minimizing penetration-induced damage to the critical Cu conductive pathways. The integration of MoS_2_ formation and subsequent OH group generation within a single PECVD chamber represents a significant process efficiency advantage. This sequentially consecutive approach eliminates wafer handling between process steps, reducing Cu-contamination risks and improving process uniformity across the process level.

**Figure 3:**
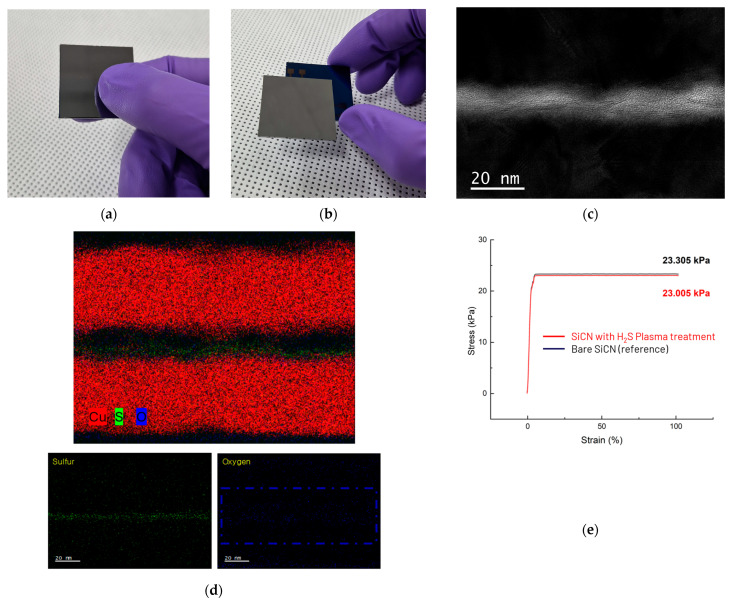
Hybrid bonding interfacial analysis. (**a**,**b**) Optical images of bonded 4 cm × 4 cm confirming successful hybrid bonding; (**c**) cross-sectional TEM image of the Cu–Cu interface after bonding; (**d**) corresponding EDS elemental maps at the bonding interface, indicating oxidation signatures of MoS_2_ at the centerline attributed to O_2_ plasma treatment; (**e**) comparison of bonding strength between SiCN processed with H_2_S plasma synthesis and pristine SiCN, showing that H_2_S plasma does not alter the intrinsic bonding mechanism or the resulting bond strength of SiCN wafer.

Following successful synthesis of the MoS_2_ contamination barrier layer on Cu surfaces, oxygen plasma treatment was performed to generate hydroxyl groups on the SiCN substrate. The process employed inductively coupled plasma (ICP) power in a PECVD system operating at 250 W under oxygen atmosphere for 30 s. After plasma treatment, the wafers were immersed in deionized water to accelerate hydroxyl group formation on the SiCN surface, thereby enhancing bonding stability. Subsequently, pattern alignment was achieved and controlled pressure was applied to establish stable hybrid bonding as shown in Figure 3a,b. The bonded structure was then annealed in a 250 °C oven for 3 h to promote Cu expansion and achieve primary metallurgical connection between the metal pads. Cross-sectional TEM imaging and EDS analysis of the Cu pads confirmed successful MoS_2_ formation between the Cu regions, with evidence of surface oxidation in the MoS_2_ passivation layer due to oxygen plasma exposure (Figure 3c,d). Tensile strength testing was conducted to verify that H_2_S plasma treatment did not adversely affect the SiCN layer, which plays a critical role in determining bonding strength. Comparative analysis between pristine SiCN samples (treated only with O_2_ plasma) and experimental samples (treated with both H_2_S and O_2_ plasma) revealed only a 1.3% difference (0.3 kPa) in vertical tensile strength. This minimal variation demonstrates that the controlled H_2_S passivation synthesis process effectively prevents Cu contamination without compromising electrical conductivity or bonding strength.

**Figure 4:**
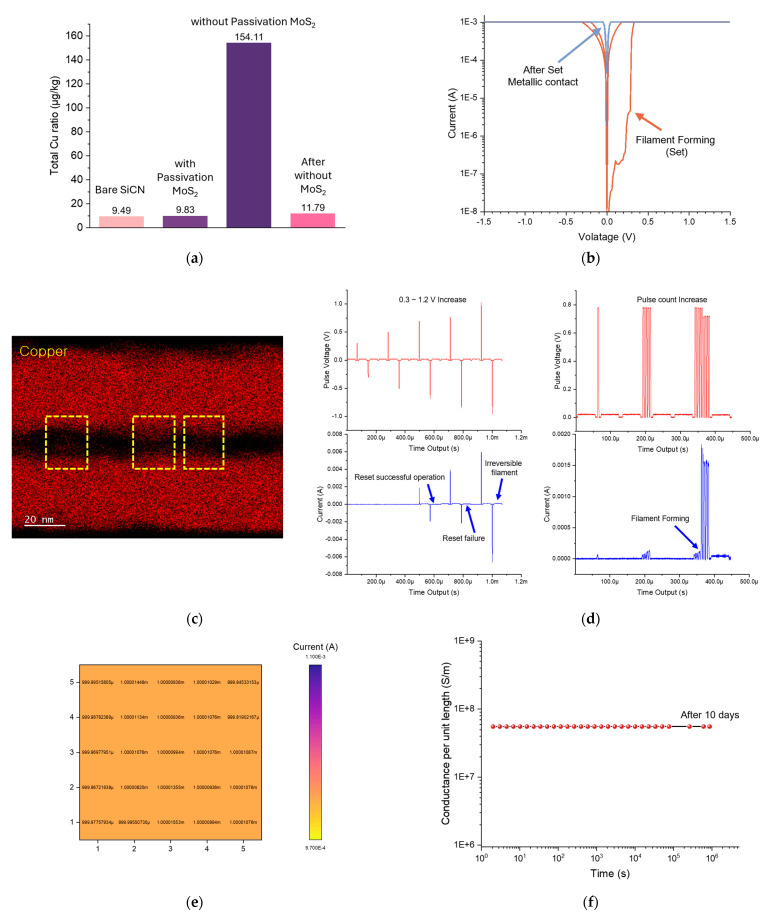
Contamination analysis and filament formation in Cu-MoS2-Cu structures. (**a**) ICP-MS quantification of Cu contamination with and without MoS2, including trend analysis under repeated processing; (**b**) post-bonding electrical forming by applying a bias across the stack to induce filament formation (Set) within MoS2 and followed by conductivity measurements; (**c**) EDS elemental mapping acquired after the Set operation, revealing Cu filaments localized within the MoS2 region. (**d**) The state of the filaments at the bonded interface was examined by analyzing the formation sites and characteristics of the filaments under various pulse conditions. The read pulse was configured to 0.02 V with a width of 50 µs, while both the Set and Reset pulses were applied with a width of 10 µs. First, a single pulse was incrementally increased in ±0.2 V steps and measured. Second, the number of pulses with an amplitude of 0.8 V and a width of 10 µs was controlled to determine the pulse count at which irreversible filament formation occurs. (**e**) This is a 5 × 5 junction mapping obtained by forming filaments through the Set operation at different 25 junction sites and subsequently measuring the resulting current flow. (**f**) Filament retention test demonstrating stable electrical connectivity attributable to the filaments even 10 days after the Set operation.

To verify the effectiveness of the synthesized MoS_2_ layer in preventing Cu contamination, SiCN coupon wafers were positioned adjacent to different sample types during O_2_ plasma treatment. The degree of Cu sputtering was quantitatively evaluated through inductively coupled plasma mass spectrometry (ICP-MS) analysis of the adjacent samples (Figure 4a). For pristine SiCN without any metallic features, the measured Cu concentration was 9.49 μg/kg, which was established as the baseline reference. When MoS_2_ was formed on Cu metal surfaces, the measured concentration was 9.83 μg/kg, representing only a 3.5% difference from the pure SiCN baseline. This minimal variation confirms that the MoS_2_ layer synthesized through the developed process successfully performs its passivation function. In contrast, when Cu metal was directly exposed during plasma treatment, ICP-MS results showed 154.11 μg/kg, representing a 15-fold increase (~1532%) compared to the baseline. This demonstrates significant Cu sputtering during O_2_ plasma processing, leading to contamination of surrounding substrates and chamber walls. To assess persistent contamination effects, a subsequent plasma process was run without any Cu present after the exposed Cu treatment, resulting in 11.79 μg/kg, which is 25% increase over the baseline. This confirms that once Cu exposure occurs during processing, continuous contamination of the chamber and adjacent substrates persist through subsequent process cycles. Beyond serving as a contamination barrier, the MoS_2_ layered structure enables it to function as a conductive medium when voltage is applied between upper and lower Cu electrodes. Cu^+^ ions migrate through grain boundaries within the MoS_2_ matrix, creating conductive pathways between the electrodes. As shown in Figure 4b, initial voltage application between the upper and lower Cu electrodes (anode and cathode configuration) performs a “Set” operation where Cu^+^ ions form Cu filaments connecting through the MoS_2_ grain boundary defect interior [35,36]. Subsequently, stable electrical connection is maintained through these Cu filaments without current loss. EDS mapping confirmed that Cu^+^ ions migrated from both upper and lower Cu electrodes formed continuous filaments, with Cu elemental distribution visible throughout the MoS_2_ interior (Figure 4c). To further elucidate the precise mechanism of filament formation and the characteristics of the resulting filaments, additional electrical pulse measurements were conducted. Single pulses with alternating polarities were sequentially applied while gradually increasing their amplitude. No notable changes were observed up to amplitudes of approximately ±0.3–0.5 V; however, at ±0.7 V, filament formation occurred successfully, followed by filament rupture at the Reset voltage. When pulses with higher amplitudes were applied, irreversible filaments formed abundantly within the relatively large Cu–MoS_2_–Cu junction area. Consequently, even under subsequent Reset voltages, these filaments did not rupture and remained stable. This behavior was leveraged to ensure reliable and robust filament formation at the bonding interface between the upper and lower samples. Furthermore, when the number of pulses (at 0.8 V) was increased, no response was observed for up to three or four pulses, whereas the application of five or more pulses led to immediate irreversible filament formation. This allowed us to identify the optimal pulse conditions for achieving stable electrical connection after bonding (Figure 4d). And also the current of 25 independently formed junctions with filaments was measured, and a 5 × 5 mapping was constructed accordingly (Figure 4e). Furthermore, these filaments maintained stable connectivity not only in the short term but also after extended periods of 1, 3, 7, and 10 days, demonstrating the capability for long-term electrical connection (Figure 4f). The controlled synthesis of MoS_2_ through the developed process effectively prevents Cu contamination during essential O_2_ plasma treatment required for hybrid bonding while simultaneously presenting a new paradigm for post-bonding electrical connectivity. This dual functionality positions MoS_2_-based barrier systems as excellent alternatives for next-generation hybrid bonding applications [21].

## 3. Conclusions

This work demonstrates a breakthrough solution to the persistent Cu contamination problem in hybrid bonding through the implementation of MoS_2_ passivation layers. The controlled PECVD sulfurization process successfully converts ultrathin Mo films to crystalline MoS_2_ at 150 °C, providing effective Cu sputtering prevention during essential O_2_ plasma treatments required for SiCN surface activation. The optimized H_2_S treatment achieves complete surface Mo conversion to MoS_2_ while preserving underlying Cu metallization integrity, as evidenced by Raman spectroscopy and cross-sectional TEM analysis. Mechanical testing reveals that the passivation process maintains SiCN bonding strength with only 1.3% variation from H_2_S plasma untreated samples, confirming preservation of fundamental bonding mechanisms essential for structural reliability. Quantitative ICP-MS analysis confirms that MoS_2_ protected samples exhibit only 3.5% contamination increase compared to pristine baselines, while exposed Cu surfaces generate 1532% contamination, demonstrating the dramatic effectiveness of the barrier approach. Beyond contamination prevention, the MoS_2_ layer enables novel memristive functionality through controlled Cu^+^ ion migration and filament formation. Post-bonding electrical connectivity is successfully established via voltage-induced Cu filament growth through MoS_2_ grain boundaries, with stable performance maintained over extended periods exceeding 10 days. The demonstrated approach eliminates chamber wall coating and cross-wafer contamination that plague conventional processes, enabling consistent manufacturing conditions and improved yield. The low-temperature processing compatibility and single-chamber integration capability position this technology for high-volume manufacturing implementation in advanced 3D integration applications. This work establishes contamination-free hybrid bonding as a viable pathway for next-generation semiconductor packaging while simultaneously introducing new paradigms for functional integration within heterogeneous systems.

## Data Availability

All data is available upon reasonable request to the authors.

## Supplementary Materials

The following supporting information can be downloaded at: https://www.mdpi.com/article/10.3390/nano15201600/s1, Figure S1: Schematic of the fabrication process. The overall schematic of the bonding process for one side of the sample is illustrated. First, pad patterns were defined on a SiCN coupon wafer using a photolithography process, followed by CCP etching to form trenches. The formed trenches were then filled with Mo/Cu using an e-beam evaporator, with the Mo layer thickness set to 2 nm. Subsequently, the photoresist was removed through acetone lift-off process, and sulfurization was carried out via PECVD to form a MoS_2_ passivation layer.
