# Supplementary material for: Cu-Contamination-Free Hybrid Bonding via MoS2 Passivation Layer
Source: Nanomaterials (Basel). 2025 Oct 21;15(20):1600. doi: 10.3390/nano15201600 (PMC12567487; doi:10.3390/nano15201600)
Supplement: Supplementary file 1 [file nanomaterials-15-01600-s001.zip › nanomaterials-3939260-supplementary.pdf]

Supplementary Figure

Cu-Contamination free Hybrid Bonding via MoS_2 Passivation Layer

Hyunbin Choi [1,†], Kyungman Kim [2,3,†], Sihoon Son [2,3,†], Dongho Lee [4], Seongyun Je [2,3], Jieun Kang [5], Sunjae Jeong [6], Doo San Kim [7], Minjong Lee [8], Jiyoung Kim [7,8] and Taesung Kim [1,2,3,4,*]

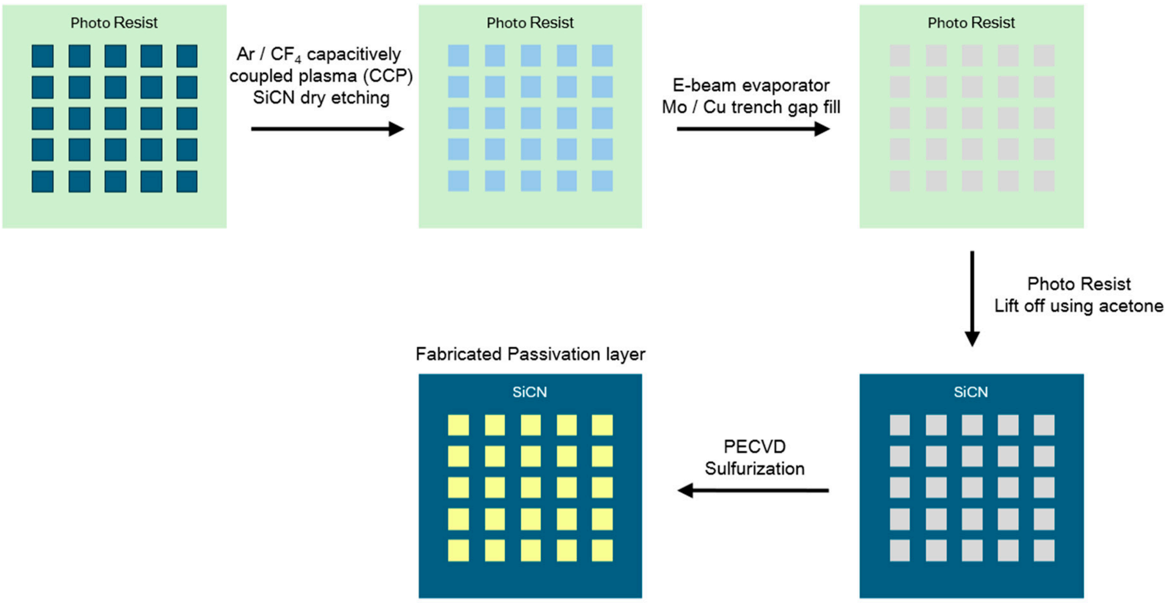

Supplementary Data Figure S1 | Schematic of the fabrication process. The overall schematic of the bonding process for one side of the sample is illustrated. First, pad patterns were defined on a SiCN coupon wafer using a photolithography process, followed by CCP etching to form trenches. The formed trenches were then filled with Mo/Cu using an e-beam evaporator, with the Mo layer thickness set to 2 nm. Subsequently, the photoresist was removed through acetone lift-off process, and sulfurization was carried out via PECVD to form a MoS_2 passivation layer.
